# RCSD1-ABL1 Translocation Associated with *IKZF1* Gene Deletion in B-Cell Acute Lymphoblastic Leukemia

**DOI:** 10.1155/2015/353247

**Published:** 2015-10-27

**Authors:** Shawana Kamran, Gordana Raca, Kamran Nazir

**Affiliations:** ^1^Shifa International Hospital, Islamabad 44000, Pakistan; ^2^University of Chicago, Chicago, IL 60637, USA; ^3^Combined Military Hospital, Kharian 50090, Pakistan

## Abstract

The *RCSD1* gene has recently been identified as a novel gene fusion partner of the *ABL1 *gene in cases of B-cell Acute Lymphoblastic Leukemia (B-ALL). The *RCSD1 *gene is located at 1q23 and *ABL1 *is located at 9q34, so that the RCSD1-ABL1 fusion typically arises through a rare reciprocal translocation t(1;9)(q23;q34). Only a small number of RCSD1-ABL1 positive cases of B-ALL have been described in the literature, and the full spectrum of clinical, morphological, immunophenotypic, and molecular features associated with this genetic abnormality has not been defined. We describe extensive genetic characterization of a case of B-ALL with RCSD1-ABL1 fusion, by using conventional cytogenetic analysis, Fluorescence In Situ Hybridization (FISH) studies, and Chromosomal Microarray Analysis (CMA). The use of CMA resulted in detection of an approximately 70 kb deletion at 7p12.2, which caused a disruption of the *IKZF1 *gene. Deletions and mutations of *IKZF1 *are recurring abnormalities in B-ALL and are associated with a poor prognosis. Our findings highlight the association of the deletion of *IKZF1 *gene with the t(1;9)(q24;q34) and illustrate the importance of comprehensive cytogenetic and molecular evaluation for accurate prediction of prognosis in patients with B-cell ALL.

## 1. Introduction

Nonrandom, acquired clonal chromosomal abnormalities are often the primary driver mutations in acute leukemia and are closely, and sometimes uniquely, associated with morphologically and clinically distinct subsets of the disease. Detection of such translocations helps in establishing the accurate diagnosis, prognosis, and risk stratification and monitoring the treatment outcomes. Translocations involving the* ABL1* gene at 9q34 are a well-known example of chromosomal abnormalities of major importance for diagnosis, risk stratification, and treatment selection in hematologic malignancies.* ABL1* codes for the protein with tyrosine kinase activity. Its major fusion partner is the* BCR* gene, involved in the t(9;22)(q34;q11) which results in a formation of the so-called Philadelphia chromosome (derivative chromosome 22). The t(9;22)(q34;q11) results in the formation of a chimeric BCR-ABL1 fusion transcript, which encodes a constitutively active ABL1 tyrosine kinase. The BCR/ABL1 fusion is a well-studied entity, present in all the cases of Chronic Myeloid Leukemia (CML) and approximately 20% cases of B-cell ALL. In addition to* BCR*, six other genes have been reported to form rearrangements with* ABL1*:* ETV6* at 12p13,* RCSD1* at 1q24,* SFPQ* at 1p34,* ZMIZ1* at 10q22.3,* NUP214* at 9q34.13,* FOXP1* at 3p12,* SNX2* at 5q23, and* EML1* at 14q32 [1–5]. However, the* ABL1* translocations with partners other than* BCR* are rare, and information about the clinical features, morphology, response to therapy, and prognosis for B-cell ALL involving such rearrangements is limited. With the development and use of targeted therapy with specific kinase inhibitors, the outcome for patients with translocations involving the* ABL1* gene has dramatically improved. Because of the likely therapeutic implications, recognizing the presence of* ABL1* fusions in newly diagnosed cases of ALL is of the highest importance.

We describe a case of B-cell ALL with the occurrence of a rare translocation involving the* RCSD1* and* ABL1* genes t(1;9)(q24;q34). Detailed clinical, morphologic, and cytogenetic correlations have only been described in the literature for two cases of B-cell ALL with confirmed RCSD1-ABL1 fusion [6, 7]. In our patient, comprehensive cytogenetic and molecular characterization including Chromosomal Microarray Analysis (CMA) allowed showing association of the RCSD1-ABL1 fusion with the* IKZF1* gene deletion, which is known as an adverse prognostic marker in B-ALL.

## 2. Materials and Methods

### 2.1. G-Banding Karyotype Analysis

Karyotypic analyses were performed on unstimulated short-term bone marrow cultures (24 and 48 hours), followed by Giemsa trypsin banding. Cytogenetic findings were described according to the International System for Human Cytogenetic Nomenclature (ISCN2013).

### 2.2. Florescence In Situ Hybridization (FISH) Analysis

FISH analysis to confirm ABL1 rearrangement was performed with the BCR/ABL1 dual color and dual fusion translocation probe (Abbott Laboratories). Additional FISH testing was done with ETV6/RUNX1-Extra signal, CEP 4-, 10-, and 17-centromeric, and MLL-Break-apart probes (Abbott Laboratories).

### 2.3. Chromosomal Microarray Analysis (CMA)

Genomic DNA was extracted with Qiagen DNeasy Blood & Tissue Kit (Qiagen Inc., Valencia, CA), according to the manufacturer's instructions. SNP array testing was performed using the Affymetrix CytoScan HD arrays (Affymetrix Inc., Santa Clara, CA) and following the manufacturer's recommendations. The data were analyzed with the Chromosome Analysis Suite (ChAS) software from Affymetrix (Affymetrix Inc., Santa Clara, CA).

## 3. Case Presentation

**Figure 1:**
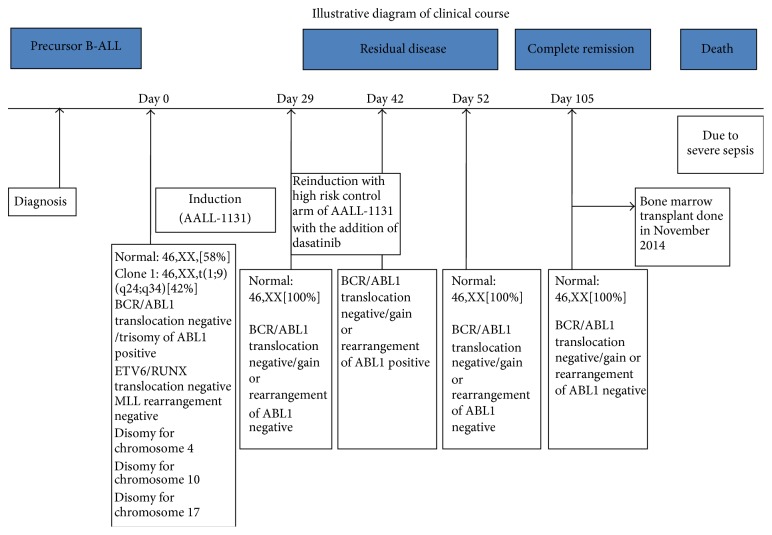
Illustrative diagram showing clinical course of the patient.

A 15-year-old female was admitted in June 2014 because of the 4-week history of sore throat and 4-day history of left thigh pain. On examination she had no hepatosplenomegaly or lymphadenopathy. Peripheral blood counts revealed hemoglobin of 9.2 g/dL, white cell count of 25100/*μ*L with 45% blasts, and platelets of 43,000/*μ*L. Bone marrow aspirate and biopsy were consistent with ALL, with the ALL-L1 FAB phenotype. Flow cytometric analysis revealed the blasts to be positive for CD19, CD34, CD10, and TdT with sCD22 and cCD79a without T-cell or myeloid antigen expression. On the basis of these findings, a diagnosis of precursor B-cell ALL was made. Cytogenetic studies revealed the karyotype 46,XX,t(1;9)(q24;q34)[10]/46,XX[14]. The patient was enrolled on AALL-1131 and received a 4-drug induction therapy with Vincristine/Daunorubicin/Pegaspargase/Prednisone. Her day 8 peripheral blood minimal residual disease (MRD) testing showed 18–25% blasts and her day 29 bone marrow evaluation revealed induction failure. She continued chemotherapy per the very high-risk control arm of AALL-1131 with the addition of dasatinib due to the* RCSD1-ABL1* fusion. Her consolidation course was complicated due to presumed fungal infection treated with Ambisome 500 mg Q 24 hours IV and C. difficile colitis treated with Metronidazole and Vancomycin PO, but she was able to complete the consolidation treatment. The last bone marrow done on November 2014 to see the remission status was hypocellular (20–25% cellularity) with regenerative trilineage hematopoiesis, mild dyserythropoiesis, and no evidence of persistent/recurrent leukemia. The patient had a bone marrow transplant from a sibling in November 2014, but a few weeks after transplant she developed severe sepsis as well as capillary leak syndrome. She remained admitted to the intensive care unit where she unfortunately died on March 12, 2015.  Figure 1 illustrates the clinical course of the disease.

## 4. Results

### 4.1. Cytogenetics

**Figure 2:**
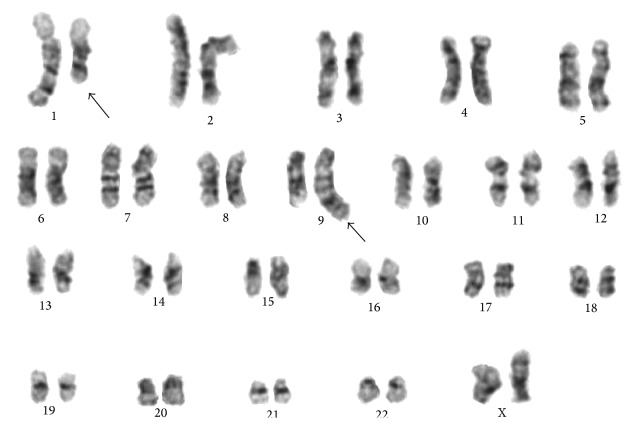
G-banded karyotype showing 46,XX, t(1;9)(q24;q34).

Cytogenetic studies done on the bone marrow sample at the time of diagnosis revealed the karyotype 46,XX,t(1;9)(q24;q34)[10]/46,XX[14] (Figure 1). The second bone marrow sample obtained after induction therapy revealed a normal female karyotype 46,XX[20] (Figure 2).

### 4.2. Florescent In Situ Hybridization (FISH)

**Figure 3:**
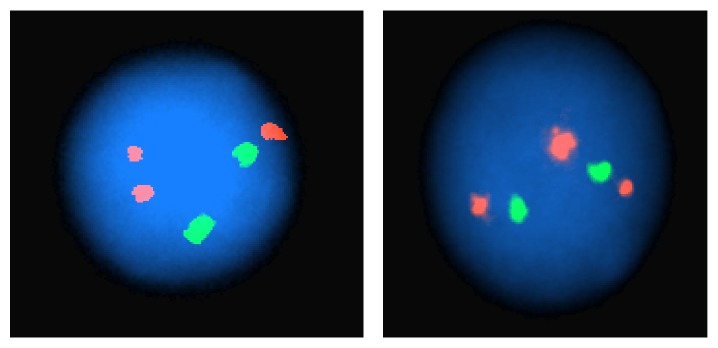
FISH at interphase revealed an extra ABL1 signal, consistent with a rearrangement of the ABL1 locus due to the t(1;9).

FISH analysis at the time of diagnosis revealed a gain of ABL1 signal in 78% of analyzed cells, consistent with the ABL1 rearrangement due to the t(1;9)(q24;q34) (Figure 3). FISH was negative for BCR/ABL1 and ETV6/RUNX1 translocations,* MLL* gene rearrangement, and trisomy for chromosomes 4, 10, and 17. BCR/ABL1 FISH testing done on bone marrow aspirate after induction therapy revealed no abnormalities. However, day 42 bone marrow aspirate showed rearrangement of the ABL1 locus, consistent with residual disease. Other consecutive samples showed negative FISH results with BCR/ABL1 probe.

### 4.3. Chromosomal Microarray Analysis

**Figure 4:**
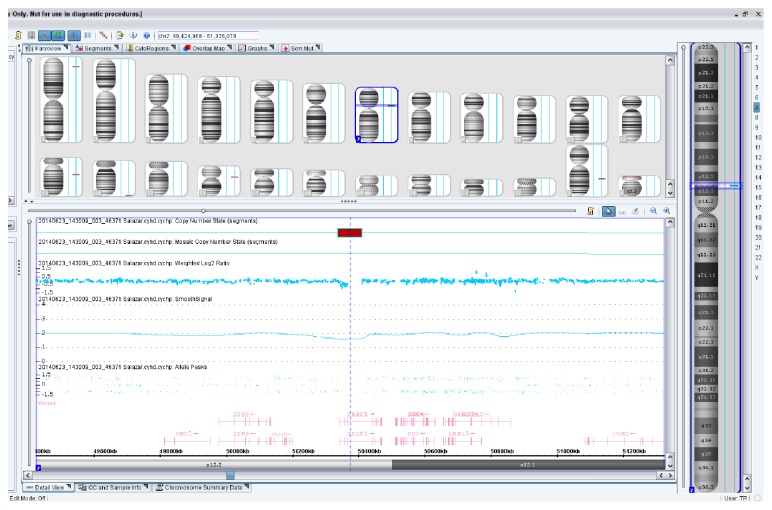
Chromosomal Microarray Analysis showing that the 70 kb deletion at 7p12.2 results in a disruption of the* IKZF1* gene.

CMA detected an approximately 70 kb copy deletion at 7p12.2 and 90 kb deletion at 13q14.2. The 70 kb deletion at 7p12.2 results in a disruption of the* IKZF1* gene (Figure 4). The 90 kb deletion at 13q14.2 results in a disruption of the* RB1* gene. The t(1;9)(q24;q34) noted by karyotype analysis was a completely balanced rearrangement and was thus not detected by CMA.

## 5. Discussion

We describe a patient with B-cell ALL associated with a particularly rare translocation affecting the* ABL1* gene, t(1;9)(q24;q34). This translocation involves the* RCSD1* gene at 1q21 and has previously been described in only four patients and confirmed by cytogenetic or molecular methods only in two patients [6–9]. The unusual feature of the RCSD1/ABL1 fusion relative to other* ABL1* rearrangements is that the N-terminal portion of the* RCSD1* gene is fused to* ABL1* starting from exon 4, while the other ABL1 fusion products also contain* ABL1* exons 2 and 3 [7]. As a consequence of the unusual breakpoint in the* ABL1* gene, the RCSD1/ABL1 protein retains only a part of the ABL SH2 domain, which would predict association of the RCSD1/ABL1 fusion with ALL rather than CML [10]. Indeed, our patient had B-cell ALL, and the same was true for the two previously reported individuals with the t(1;9). The* RCSD1* gene codes for a protein kinase substrate CapZIP (CapZ-interacting protein), which is highly expressed in skeletal muscle and more weakly expressed in cardiac muscle. It has been shown that stress-induced phosphorylation of CapZIP may regulate the ability of CapZ to remodel actin filament assembly which is an important step in mitosis [11]. As* ABL1* protein has a tyrosine kinase activity and its fusion proteins act as constitutively activated tyrosine kinases, the formation of the* RCSD1-ABL1* fusion gene could therefore result in CapZIP phosphorylation with loss of interaction between CapZIP and CapZ, leading to an alteration of the cellular function by affecting the cytoskeleton regulation, which could be an important step in leukemogenesis. It is important to note that B-ALL with the RCSD1-ABL1 fusion has recently been included into a specific molecular subtype of high-risk B-ALL, known as Philadelphia- (Ph-) like ALL, which is characterized by an expression signature resembling that of Philadelphia-positive ALL [12]. Similar to Ph-positive cases, these “Ph-like” leukemia cases have a high probability of relapse and carry poor prognosis, but it has been postulated that they may respond to treatment with tyrosine kinase inhibitors (TKIs), since they are characterized by activated kinase signature. Indeed, in vitro and in vivo studies, as well as increasing number of case reports, have documented efficacy of using targeted TKIs for Ph-like leukemia cases [12–14], but the prognostic significance of this fusion is still unclear. The patient described by De Braekeleer et al. [2] responded well to initial therapy and achieved a complete remission but relapsed 2 years after the initial treatment.

The patient reported by Mustjoki et al. [7] received a modified CVAD induction therapy and, after two induction courses, still had a residual disease. However, the patient achieved morphological remission after addition of a wide-spectrum TKI dasatinib. Similarly, the patient described by Inokuchi et al. did not achieve complete remission with hyper-CVAD combination chemotherapy but achieved transient clinical effects with TKIs, imatinib, and dasatinib, coadministered with dexamethasone. The patient described in this report did not respond to the standard induction chemotherapy and only achieved full morphologic and cytogenetic remission with continued chemotherapy per the very high-risk control arm of AALL-1131 with the addition of dasatinib. Long-term follow-up information is unfortunately not available, but our case supports previous observations that B-ALL with the RCSD1-ABL1 fusion may be associated with increased risk of induction failure and is also characterized by susceptibility to TKI treatment. In addition to induction failure, a high risk of relapse has also been described in ALL with RCSD1-ABL1 fusion. In the case reported by Inokuchi et al., after short remission on TKIs, the leukemic cells rapidly became refractory to the TKI treatment and subsequently developed three additional reciprocal chromosomal translocations, t(5;16)(q33;q24), dic(18;20)(p11.2;q11.2), and t(10;19)(q24;p13.3). This led the authors to hypothesize that the B-ALL with the RCSD-ABL1 fusion may be characterized by high genomic instability and propensity for clonal evolution and development of resistance.

Other factors that may modify the disease course and outcome in patients with RCSD1-ABL1 fusion are associated secondary genetic changes, including mutations and deletions of the* IKZF1* gene.* IKZF* codes for a DNA-binding protein Ikaros which displays crucial tumor suppressor functions in the hematopoietic system. Loss of Ikaros function has been linked to the development of lymphoid leukemia, in particular precursor B-ALL, and has been shown to confer a poor treatment outcome. Somatic aberrations of* IKZF1* have been observed with particularly high frequency in B-ALL cases carrying a BCR/ABL1 rearrangement. More recently, it has also been demonstrated that cases of Ph-like ALL frequently carry* IKZF1* abnormalities [12, 13]. Despite the associations with other high-risk genetic abnormalities* IKZF1* deletions still represent an independent predictor of poor prognosis [13]. Combined use of different cytogenetic and molecular techniques allowed us to detect cooccurrence of the RCSD1-ABL1 fusion and* IKZF1* deletion in our patient. No clinical correlates exist for this rare association to date. Although long-term follow-up information is not available for this case, we anticipate that the simultaneous presence of two high-risk genetic abnormalities in leukemia cells (RCSD1-ABL1 fusion and* IKZF1* deletion) would predict a very high risk of relapse.

Our findings illustrate the importance of comprehensive genetic evaluation using a variety of assays in order to achieve fully accurate risk stratification for B-ALL patients. Additional cases will have to be identified and studied to ascertain the prognostic significance of the RCSD1-ABL1 fusion with and without cooccurrence of* IKZF1* abnormalities. Additionally, the effectiveness of combined treatment with chemotherapy and TKIs for patients with the RCSD1-ABL1 fusion and other genetic lesions associated with Ph-like ALL will have to be explored through large multicenter clinical trials.
